# Hematological parameters of reproductive-age women using hormonal contraceptives at University of Gondar Comprehensive Specialized Referral Hospital, Northwest Ethiopia: A comparative cross-sectional study

**DOI:** 10.1371/journal.pone.0277254

**Published:** 2022-11-08

**Authors:** Solomon Gedfie, Solomon Getawa, Woldeteklehaymanot Kassahun, Kiros Terefe Gashaye, Mulugeta Melku

**Affiliations:** 1 Department of Medical Laboratory Sciences, College of Medicine and Health Sciences, Woldia University, Woldia, Ethiopia; 2 Department of Hematology and Immunohematology, School of Biomedical and Laboratory Sciences, University of Gondar, Gondar, Ethiopia; 3 Department of Obstetrics and Gynecology, School of Medicine, College of Medicine and Health Sciences, University of Gondar, Gondar, Ethiopia; Oregon State University, UNITED STATES

## Abstract

**Background:**

More than one-third of reproductive aged women in Ethiopia use hormonal contraceptives to prevent conception. The present study aimed to compare the hematological parameters of reproductive-age women taking hormonal contraceptives at the University of Gondar Comprehensive Specialized Referral Hospital, Northwest Ethiopia in 2021.

**Methods:**

A comparative cross-sectional study was conducted from April to June 2021. A total of 240 study participants were recruited by using a consecutive sampling technique. Data on socio-demographic variables and clinical data were collected through face-to-face interviews using a structured questionnaire and medical record reviews, respectively. Three milliliter venous blood was collected for complete blood count analysis using Unicel DxH 800 coulter hematology analyzer. Data was entered into Epi-data 4.4.3.1 version then exported to IBM SPSS v25 for analysis. Kruskal-Wallis H, Dunn-Bonferroni pairwise comparison test, and Spearman’s correlation analysis were used for inferential statistics. P<0.05 were considered statistically significant.

**Result:**

The median and interquartile range of platelet count among combined oral contraceptive users was 285(238–332) which is significantly higher than that of depot medroxyprogesterone acetate users 246(220–226) (p = 0.010), implant user 247(221–297) (p = 0.034), and controls 256(224–278) (p = 0.015). The result also showed long-term use of implant negatively correlated with red blood cell count (p = 0.033).

**Conclusion:**

This finding concludes that combined oral contraceptive users had a higher platelet counts than controls while long-term use of implants can result in low red blood cells count. Therefore, a baseline evaluation of complete blood count in women desiring contraceptive methods would also be recommended.

## Introduction

Contraception is the deliberate avoidance of conception by using various devices, sexual practices, chemicals, drugs, or surgical techniques [1]. Hormonal contraceptives (HCs) are synthetic biological chemicals that have been used to prevent unwanted pregnancy [2].

Globally, the use of modern contraception has risen slightly, from 54% in 1990 to 57.4% in 2015 while in Africa it went from 23.6% to 28.5%. In Ethiopia the prevalence of contraceptive use among reproductive women was reported as 37.6%. Out of these 57.0%, 24.3%, and 7.1%, used Depot medroxyprogesterone Acetate (DMPA), implant, and Oral contraceptive pills (OCPs) users, respectively [3]. In Gondar town the magnitude of contraceptive utilization is about 41.2% which is nearly half of the reproductive-age women, the majority of them use hormonal-based contraceptives [4].

Users’ interest in the safety and efficacy of HCs has grown over time. The majority of the concern has been about the impact of estrogen and progesterone constituents of HCs on various biochemical and physiological processes of users [5]. Scientific evidence indicates that HC changes binders of vitamin B_12_ such as transcobalamin I and transcobalamin III [6]. In the serum and plasma, it is associated with alterations in the absorption of some trace elements and vitamins which are vital for blood cell production [7, 8]. Studies showed that estrogen administration can initiate the division and proliferation of Hematopoietic Stem Cells (HSCs) and thus explains the higher blood counts in women during the reproductive years. However, in some family planning clinics, women taking hormonal contraceptives face the problem of irregular vaginal bleeding, amenorrhea, and sometimes excessive bleeding which could lead to anemia [9, 10].

Prolonged use of progestin-only contraceptives can be an aggravating factor for the development of anemia through irregular and heavier bleeding patterns [11]. Long-term use of HC can influence mean cell volume (MCV), mean cell hemoglobin (MCH), and red cell distribution width (RDW) causing macrocytic effect on the red blood cells (RBC) since it affects vitamin B12 and folate absorption [12, 13]. Studies showed that OCPs use could increase platelet count [14]. HC usage could also be associated with decreased percent packed cell volume(%PCV), WBC count, percent lymphocyte, and increased platelet count [15]. Besides, HC might increase RBC count as compared to the non-users but long-term usage of HC could have a negative effect on RBC count [16]. Despite the benefit that contraceptive use may have with respect to maintain the blood cells, little data was available in the study area on the effect of contraceptive methods on hematological parameters. Furthermore, some of the studies offered had flaws such as a lack of appropriate comparison groups or were too small to draw meaningful conclusions.

In general, these commonly used HC methods are well-tolerated, but given their long-term and frequent use, even rare adverse effects and complications have generated significant public concern. In Ethiopia, even though it has a significant health impact, there is no epidemiological evidence published in the area related to the hematological profiles of HC users. Therefore, the present study aimed to compare the hematological parameters of HC user reproductive-age women with apparently healthy non-user controls at the University of Gondar Comprehensive Specialized Referral Hospital.

## Methods and materials

### Study area, design and period

A comparative cross-sectional study was conducted at the family planning clinic of the University of Gondar Comprehensive Specialized Referral Hospital, Northwest Ethiopia. The hospital is located in the Central Gondar zone, Gondar town administration. The town is located in Amhara regional state, 727 km far from Addis Ababa, the capital city of Ethiopia. The family planning clinic deliver services such as antenatal care, post-natal care, and contraceptive services. This study was conducted from 01 April to 30 June 2021.

### Inclusion and exclusion criteria

#### Inclusion criteria

For HC user groups, HC user reproductive-age women and who were taking HCs (injectable DMPA, Implant, or COC) for at least six months as a contraceptive during the study period were included in the study. For the control group, reproductive-age women who visited the family planning clinic and were not taking any contraceptive at least for the past six months and who were volunteers to participate were included in the study.

#### Exclusion criteria

Lactating women, pregnant women, women who had a history of chronic illness like diabetes mellitus, hypertension, kidney disease, cardiac disease, human immunodeficiency virus (HIV), hepatitis, and critically ill women during the study period were excluded from the study for all groups. Information regarding their history of infection was collected and all study participants were screened for the presence of active infections using laboratory methods appropriate for the investigation. To screen women whether they have parasitic infection; stool, urine and peripheral blood examinations were performed. Regarding the screening of viral infections like HIV and Hepatitis, rapid diagnostic test algorisms such as SD BiolineTM tri-line HIV-1/2/0 Rapid test kit and Hepatitis B surface antigen test kit and Hepatitis C antibody qualitative detection test kits were used. After evaluation of the result of the tests performed, we have excluded those women with positive results in any one of the tests performed.

#### Sample size determination and sampling technique

According to rules of thumb that have been recommended by van Voorhies and Morgan, 30 participants per group are required to recognize real differences, which could achieve an estimated 80% power [17]. To maximize the power of the study, the number of study participants was increased two-fold for each group. Thus, a total of 240 (60 for DMPA, 60 for Implant, 60 for COC, and 60 for controls) study subjects were enrolled in the study. The study participants were selected using a consecutive sampling method by assuming that, participants flow to the clinic was random.

### Operational definition

- **Reproductive-age women**: women in the age group of 15–49 years [18].
- **Hormonal contraceptive user:** women using among any of the three contraceptives like DMPA, implant, or COC as a mechanism of birth control [19].
- **Implant users**: women who use a small flexible plastic rod that is placed under the skin in their upper arm by a doctor or nurse for at least six months, it contains 68 mg of Etonogestrel [19].
- **COC user**: women uses orally designed birth control pills for at least six months, it is a combination of an estrogen) (Ethinylestradiol (EE) (0.03mg)) and a progestogen (0.15mg levonorgestrel +75 mg ferrous sulfate) [19].
- **DMPA user**: women use an injectable contraceptive injected with a syringe in the muscle or the fatty tissue for at least six months, it contains 150mg/ml of medroxyprogesterone acetate (medroxyprogesterone acetate 400mg, polyethylene glycol 20.3mg, and sodium sulfate anhydrous 11mg) [19].
- **Controls**: women did not use contraceptives at least for the past six months.

### Data collection and data collection procedures

#### Socio-demographic and clinical data collection

Data on socio-demographic characteristics of the women like age, residence, and clinical characteristics of the women like the type of contraceptive and duration of use were collected using pretested structured questionnaires via face-to-face interview and using a checklist from medical records by Midwives, respectively.

### Laboratory sample collection and analysis

#### Blood sample collection

Following the standard operating procedures (SOPs), 3 ml of venous blood was collected by an experienced laboratory technologist from the medial cubital vein of the left arm using a vacutainer tri-potassium ethylene tri-amine tetra-acetic acid (K_3_EDTA) tube after cleaning the venipuncture site with 70% alcohol. The sample was mixed with the anticoagulant properly by inverting two to three times.

#### Complete blood count test

The laboratory analysis was done at the University of Gondar Comprehensive specialized hospital hematology laboratory room by Unicel® DxH 800 Coulter cellular analysis (Beckman Coulter, Ireland), which is a quantitative automated hematology analyzer for in vitro diagnostic use in screening patients populations found in clinical laboratories. The UniCel® DxH 800 analyzer provides a CBC, leukocyte 5 part differential, reticulocyte, and nucleated red blood cell on whole blood [20].

#### Data management and quality control

The questionnaire was prepared in English and translated to the local language, Amharic, and retranslated back to English to check the consistency. Pretest was done on 5% of the study participants to ensure the validity of the questionnaires. Additionally, training was given to the data collectors (midwives and laboratory technologists) about the objective and relevance of the study, confidentiality issues, study participants’ rights, techniques of interview, and result recording. The Pre-analytical, analytical, and post-analytical phases of quality assurance were maintained during sample processing by carefully following standard operating procedures (SOPs). In the pre-analytical phase, the quality was assured during patient identification, labeling, sample collection, sample transportation, and storage by careful adherence to SOPs. The quality control (QC) for working equipment and reagents was ensured by running low, normal, and high QC materials. Post analytical quality assurance methods were undertaken during result interpretation, reporting, and recording. To ensure quality, the collected data were checked out for completeness, accuracy, and clarity by data collection supervisors and the principal investigator.

### Data analysis and interpretation

Data were coded and entered into EpiData version 4.4.3.1 and then exported to SPSS (IBM Corporation, Armonk, NY, USA) version 25 software for analysis. Homogeneity of variance was checked using Levene’s statistics. The Shapiro-Wilk normality test was used for checking the distribution of continuous variables, and it revealed that the variables were not normally distributed for the group. Therefore, non-parametric tests, Kruskal-Wallis H, and Dunn-Bonferroni pairwise comparison were used for the comparison of hematological parameters between groups. Spearman correlation coefficient was used to determine the correlation between duration of contraceptive use and hematological parameters for non-parametric outcome variables. The results were presented as median and Interquartile Range (IQR). Summarized data were presented with texts and tables. A P-value less than 0.05 was considered statistically significant.

### Ethical considerations

The study was approved by the Ethical Review Committee of the School of Biomedical and Laboratory Sciences, College of Medicine and Health Science, the University of Gondar with an ethical clearance number SBMLS-2791. A permission letter to conduct the study was also obtained from the University of Gondar Comprehensive Specialized Referral Hospital’s chief clinical director’s office. Besides, written informed consent was obtained from the study participants while informed assent and informed consent was obtained from participants aged 15 to 17 years and their parents or guardians respectively. All the information obtained from the study participants was kept confidential. Laboratory test results were communicated to the responsible clinicians working at the University of Gondar Comprehensive Specialized Referral Hospital, family planning clinic for further treatment of participants having abnormal results.

## Result

### Socio-demographic characteristics

A total of 240 reproductive-age women who were attending the University of Gondar Comprehensive Specialized Referral Hospital family planning clinic from April to June 2021 were included in the study. The study participants were categorized into four groups: DMPA users, implant users, COC users, and non-contraceptive users. Each group consisted of 60 individuals. The mean age of the subjects was 26 (26.73±4.93) years. Of the enrolled individuals, the majority of them, 218 (90.8%) were Orthodox Christians and 230 (95.8%) were urban residents. Of the participants, 171 (71.3%) were married and 85 (35.4%) were housewives in their occupation. Regarding their educational status, 87 (36.3%) were attended college and above (Table 1).

**Table 1:** Socio-demographic characteristics of hormonal contraceptive users and non-users at University of Gondar Comprehensive Specialized Referral Hospital, Northwest Ethiopia, 2021(n = 240).

Socio-demographic variables		Frequency	Percentage (%)
Place of residence	Urban	230	95.8
	Rural	10	4.2
Marital status	Single	65	27.1
	Married	171	71.3
	Divorced	4	1.7
Occupational status	Student	63	26.3
	Housewife	85	35.4
	Government employee	29	12.1
	NGO employee	17	7.1
	Merchant	26	10.8
	Others	20	8.3
Educational status	unable to read and write	20	8.3
	Primary	55	22.9
	Secondary	78	32.5
	College and above	87	36.3
Age group (years)	15–24	85	35.4
	25–34	132	55.0
	35–49	23	9.6

NGO = Non-Governmental Organization,

### Duration of contraceptive use

**Fig 1:**
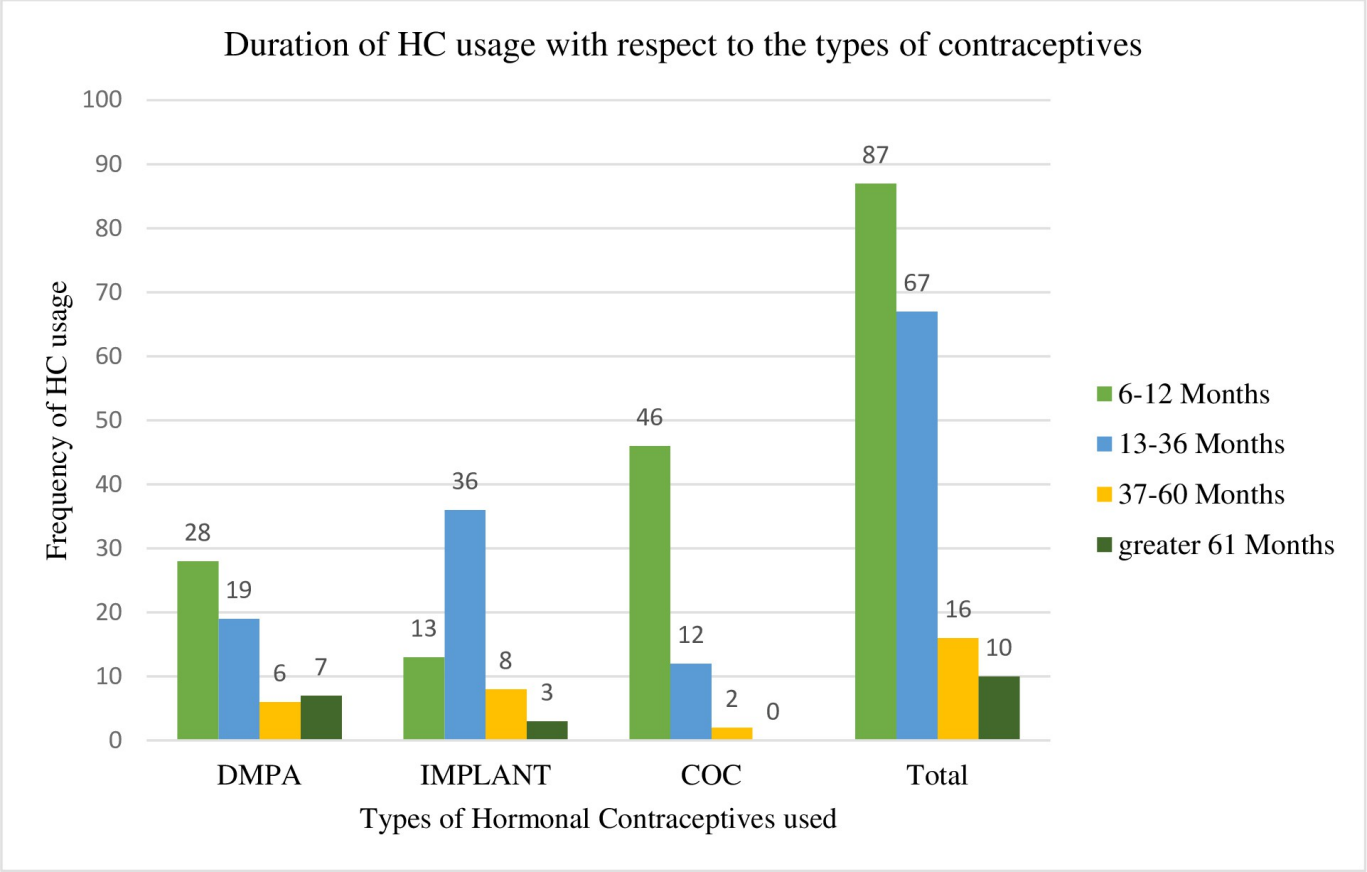
Duration of HC usage with respect to the types of contraceptives at University of Gondar Comprehensive Specialized Referral Hospital, Northwest Ethiopia, 2021 (n = 180). DMPA = Depotemedroxy progesterone Acetate, COC = combined oral contraceptive.

The majority of the DMPA users use it for 6–12 months duration while the majority of the implant users use 13–36 months duration and that of the COC users were majority use 6–12 months (Fig 1).

### Comparison of hematological parameters

**Fig 2:**
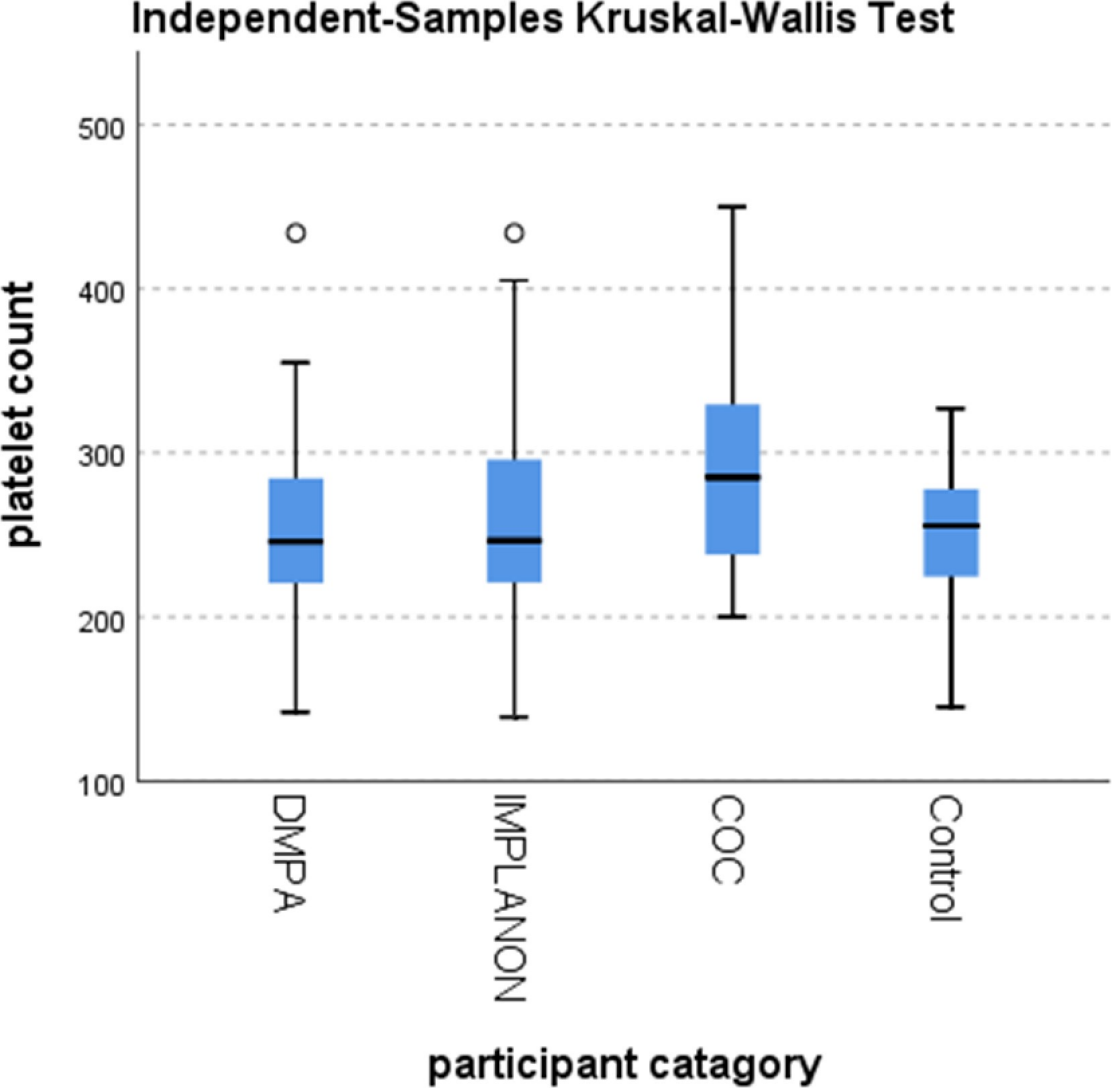
Independent samples Kruskal-Wallis test box plot of platelet count distribution across the participant categories.

Since the data were not normally distributed across the groups, non-parametric tests (Kruskal-Wallis H test followed by post-hoc-Bonferroni pairwise comparison test) were used to compare the median differences in hematologic parameters between study groups (Fig 2). Platelet count was highest in COC users followed by controls as compared to DMPA and implant users. In Kruskal-Wallis analysis, the median and IQR of platelet count was 285 (238–332) x10^9^/L, 246 (220–286) x10^9^/L, 247 (221–297) x10^9^/L, and 256 (224–278) x10^9^/L among COC, DMPA, IMPLANON users and non-contraceptive users (controls), respectively. Platelet counts were found significantly higher in COC users than in other groups (p = 0.004). There was no statistically significant difference between the median and IQR of other hematological parameters between the groups (Table 2).

**Table 2:** Comparison of the hematological profile of HC users and non-users at University of Gondar Comprehensive Specialized Referral Hospital, Northwest Ethiopia, 2021 (Median, IQR, and p-value) (n = 240).

Parameter	Category of participant				p-value
	DMPA(n = 60)	Implanon(n = 60)	COC(n = 60)	NCU/control(n = 60)	
	Median (IQR)	Median (IQR)	Median (IQR)	Median (IQR)	
RBC×10^6^/mm^3^	4.82(4.43–5.20)	4.81(4.53–5.05)	4.85(4.51–5.03)	4.76(4.45–5.09)	0.899
HGB (g/dl)	14.20(13.45–15.08)	14.10(13.60–15.00)	14.20(13.40–14.88)	14.00(13.33–14.78)	0.615
HCT (%)	42.8(39.9–45.3)	42.7(40.9–45.5)	43.0(40.7–44.5)	43.0(40.0–44.4)	0.891
MCV (fl)	89.1(86.6–92.2)	89.2(86.7–91.9)	89.6(86.1–91.8)	89.1(86.0–91.2)	0.955
MCH (pg)	29.8(28.6–30.8)	29.8(29.0–30.9)	29.8(28.4–30.6)	29.2(28.6–30.9)	0.613
MCHC (g/dl)	33.2(32.6–33.7)	33.2(32.7–33.8)	33.3(32.9–33.6)	33.2(32.6–33.7)	0.900
RDW (%)	13.6(13.0–14.2)	13.7(13.2–14.5)	13.5(13.0–14.2)	13.6(13.0–14.4)	0.547
PLT ×10^9^/l	246(220–286)	247(221–297)	285(238–332)	256(224–278)	0.004*
MPV (fl)	9.3(8.2–10.0)	9.2(8.2–10.3)	8.7(7.9–9.8)	9.0(8.4–9.9)	0.441
WBC ×10^9^/l	5.7(4.3–7.0)	6.0(4.7–7.1)	6.2(4.8–7.7)	6.3(5.1–7.8)	0.197
NEU (%)	54.4(43.0–60.6)	50.1(40.4–56.1)	52.2(43.4–58.8)	53.0(43.4–60.4)	0.374
LYM (%)	32.8(28.6–42.6)	38.0(31.1–43.5)	34.2(28.5–41.3)	35.8(27.3–43.2)	0.341
MON (%)	6.4(5.0–8.0)	6.5(5.2–8.2)	6.6(5.3–8.0)	7.2(5.5–8.3)	0.665
EOS (%)	3.0(1.6–6.1)	3.2(1.5–7.0)	3.9(2.1–8.3)	2.6(1.4–6.6)	0.202
BAS (%)	0.9(0.7–1.1)	0.9(0.7–1.1)	0.7(0.5–1.1)	0.8(0.5–1.1)	0.092

DMPA = Depotemedroxy progesterone Acetate, COC = combined oral contraceptive, NCU = Non Contraceptive User, RBC = red blood cell, Hb = hemoglobin, MCH = Mean Cell Hemoglobin, MCHC = Mean Cell Hemoglobin Concentration, MCV = Mean Cell Volume, MPV = Mean Platelet Volume, RDW = Red cell distribution width, WBC = White blood cell, PLT = Platelet, NEU (%) = Neutrophil percent, LYM (%) = Lymphocyte percent, MON (%) = Monocyte percent, EOS (%) = Eosinophil percent, BAS (%) = Basophil percent, IQR = Interquartile range,

* = significant at p-value <0.05.

### Multiple pairwise comparisons of platelet count

Multiple pairwise comparisons of platelet count were done to know at which group the specific difference of the median (IQR) of platelet count happened. The test showed that platelet count in the COC user group was significantly higher than the rest three groups (p-value <0.004) (Table 3). In Bonferroni pairwise comparison tests between groups, there were a statistically significant higher median value of platelet count in COC group compared with DMPA (p-value = 0.01), Implant (p-value = 0.034) and control groups (p-value = 0.015) (Table 4).

**Table 3:** Pairwise comparisons of platelet count across the groups using Kruskal-Wallis H test (post-hoc-Bonferroni pairwise comparison tests) at University of Gondar Comprehensive Specialized Referral Hospital, Northwest Ethiopia, 2021(n = 240).

Group 1-Group 2	Test Statistic (H-test)	Std. Error	Std. Test Statistic	Sig.	Adj. Sig.^a^
DMPA**-Control***	-1.667	12.675	-0.131	0.895	1.000
DMPA**-IMPLANON***	-4.892	12.675	-0.386	0.700	1.000
DMPA**-COC***	-40.008	12.675	-3.157	0.002*	0.010*
Control***-IMPLANON**	3.225	12.675	0.254	0.799	1.000
Control***-COC**	38.342	12.675	3.025	0.002*	0.015*
IMPLANON**-COC***	-35.117	12.675	-2.771	0.006*	0.034*

Std.Error = standard error, Std. Test Statistic = standard Test Statistic, Sig. = significance, Adj. Sig, = adjusted significance, COC = combined oral contraceptive, DMPA = Depotemedroxy progesterone Acetate,

* = significant at p-value <0.05,

** = group 1,

*** = group 2

**Table 4:** Correlation of duration of contraceptive use with hematological parameter change at University of Gondar Comprehensive Specialized Referral Hospital, Northwest Ethiopia, 2021 (n = 180).

Hematologic parameter vs duration of contraceptive use	Contraceptive category		
	DMPA	Implant	COC
	rho (ρ), p-value	rho (ρ), p-value	rho (ρ), p-value
RBCx10^6/^mm^3^	-0.046, (0.725)	-0.276, (0.033)*	0.151, (0.250)
Hgb(g/dl)	0.094, (0.473)	-0.205, (0.116)	0.180, (0.169)
Hct(%)	0.044, (0.741)	-0.214, (0.101)	0.187, (0.153)
MCV(fl)	0.036, (0.783)	0.096, (0.465)	-0.051, (0.696)
MCH(pg)	0.062, (0.635)	0.127, (0.332)	-0.067, (0.610)
MCHC(g/dl)	0.060, (0.647)	0.037, (0.778)	0.021, (0.872)
RDW(%)	-0.062, (0.638)	-0.078, (0.556)	0.047, (0.724)
PLTx10^9^/l	0.119, (0.366)	-0.090, (0.492)	0.065, (0.624)
MPV(fl)	-0.112, (0.396)	-0.127, (0.335)	0.023, (0.860)
WBCx10^9^/l	0.061, (0.641)	-0.046, (0.727)	-0.053, (0.688)
NEU(%)	0.044, (0.739)	-0.003 (0.981)	0.011, (0.934)
LYM(%)	-0.094, (0.475)	0.003, (0.984)	0.182, (0.163)
MON(%)	-0.091, (0.491)	-0.155, (0.235)	-0.135, (0.303)
EOS(%)	-0.008, (0.952)	0.019, (0.884)	-0.133, (0.313)
BAS(%)	-0.177, (0.177)	0.180, (0.169)	-0.075, (0.570)

DMPA = Depotemedroxy progesterone Acetate, COC = combined oral contraceptive, RBC = red blood cell, Hb = hemoglobin, MCH = Mean Cell Hemoglobin, MCHC = Mean Cell Hemoglobin Concentration, MCV = Mean Cell Volume, MPV = Mean Platelet Volume, RDW = red cell distribution width, WBC = white blood cell, PLT = platelet, NEU (%) = Neutrophil Percent, LYM (%) = Lymphocyte percent, MON (%) = monocyte percent, EOS (%) = eosinophil percent, BAS (%) = basophil percent, MPV = mean platelet volume,

* = significant at p-value <0.05.

### Correlation analysis of duration of contraceptive use

Spearman’s correlation analysis of hematological parameters and duration of contraceptive use showed RBC count (p = 0.033) were negatively correlated with implant usage (Table 4).

## Discussion

This comparative cross-sectional study tried to compare the hematological parameters of hormonal contraceptive users with the non-users. Correlation analysis was also done between the duration of contraceptive use with hematological parameter changes. Previous studies showed different patterns of hematological parameters among different HC users. Progestin-only hormonal preparations are associated with a predisposition to the risk of thromboembolism with the involvement of platelets [5]. The menstrual irregularities were found to be more frequent in users of injectable HCs compared to non-users, especially amenorrhea and irregularities of menstrual flow. In users of COC, hemoglobin and hematocrit were slightly better maintained as compared to non-users [5].

Oral contraceptive use may cause changes in the hemostatic system. Estrogen could be a risk factor for the development of thromboembolic disease [21]. The results of this study showed an increase in the median (IQR) of platelet count in COC users compared to the control group and demonstrated that there was a significant increase in platelet count in oral contraceptive users as compared to the controls group (P = 0.015). This finding is in agreement with studies done in Netherland, Sudan, Al-Samawah (Iraq), and Bangladesh [14, 22–24]. However, the finding is on the contrary with a study done in Kaduna State, Nigeria [9]. which reported decreased platelet count. The variation may be due to differences in geographical location, variation in comparison method, and differences in way of life of the study participants.

Our finding showed that COC users had significantly higher values of platelet count compared to DMPA and Implant users (p = 0.010). The finding is in agreement with a study done in Karachi, Pakistan and Kaduna State, Nigeria [9, 25]. This could be due to a decrease in menstrual blood loss with episodes of amenorrhea in COC users than the DMPA and Implant users. It may also be due to the menstrual irregularity caused by the injectables [26]. and Long-term use of implant contraceptives leads to suppressed production of blood cells [11].

The findings of this study showed a statistically significant negative correlation between the duration of implant use with RBC count. This finding is in agreement with a study was done in Iraq [11]. who reported long-term implant use had a higher risk of anemia compared to short-term use. This is due to decreased intestinal reabsorption of folate, which resulted in decreased serum folate levels [27, 28]. Implant users also experienced increased and prolonged menstruation which resulted in blood loss [29]. The finding of this study is on the contrary with a study done in Nasr City, Egypt [30]. who reported that long-term use of implants was of benefit against anemia. The variation may be due to the difference in geographical location, variation in sample size, and duration of contraceptive use differences [30].

The findings of the present study showed that the median (IQR) of RBC count and Hb level did not show a significant difference between the groups. The report in this study disagrees with the studies conducted in Pakistan and Canada [11, 31]. who reported lower RBC count and Hb level and studies conducted in Nasr City, Egypt, and Sokoto State North-Western Nigeria [10, 30]. respectively, which reported higher RBC count and Hb level than the controls. Also, there is no statistically significant change in the Hct (%) values between the groups, which is in contrast with the study conducted in Al-Samawah city, Iraq [23]. who reported lower values of Hct (%) compared to the controls. The variation could be due to differences in socio-demographic locations, socio-economic status, duration of contraceptive use, and ways of life [32].

The results of the present study showed that the median total WBC count of the HC user and controls did not show a significant difference. This finding was in contrary the study conducted in Al- Samawah city Iraq [23]. which reported an increase in total WBC count. There is no significant difference in the percentage of neutrophils, lymphocytes, monocyte, eosinophil, and basophil in this study. However, studies conducted in Al- Samawah city Iraq, reported a lower percentage of lymphocyte count and granulocyte count [23]. The variation might be due to differences in duration of contraceptive use, geographical location, and variations in sample size [33].

The first limitation of this study was the cross-sectional nature of the study design that does not tell us which appeared first, either HC use or hematological parameter changes. Moreover, the study was done in single centered area, this may potentially limit the generalizability of the result. This study is also limited for not assessing the coagulation parameters.

## Conclusion and recommendation

The findings in this study showed there is a statistically significant increase in platelet count among COC users compared to the controls, DMPA, and implant users. On the other hand, there was a statistically significant negative correlation between long-term use of implant contraceptives and RBC count. This suggests that COC use may increase the platelet count.

Therefore, women suffering from low-platelet-count may benefit from using COC, so the non-contraceptive benefits of COC should be considered for those desiring contraceptives. However, in places where anemia is common, long-term use of implants may add more morbidity due to decreased RBC production. Accordingly, healthcare givers can always weigh the benefits of the hormonal contraceptive use against their side-effects and should guide when to stop to prevent other adverse effects. However, further studies with a large scale are required to elucidate these issues. Baseline evaluation of CBC in women before using the HC methods is also recommended.

## Supporting information

S1 Dataset The minimal data set used to synthesis the results of this study. (SAV)

## Abbreviations

ANOVA: Analysis of Variance
CBC: Complete Blood Count
COC: Combined Oral Contraceptive
DMPA: Depot medroxyprogesterone Acetate
EDTA: Ethylene Diamine Tetra Acetic Acid
Hb: Hemoglobin
HCG: Human Chorionic Gonadotropin
HCs: Hormonal Contraceptives
IUD: Intrauterine Device
MCH: Mean Cell Hemoglobin
MCHC: Mean Cell Hemoglobin Concentration
MCV: Mean Cell Volume
MPV: Mean Platelet Volume
OC: Oral Contraceptive
OCPs: Oral Contraceptive Pills
OR: Odds Ratio
PCV: Packed Cell Volume
RBC: Red Blood Cell
RDW: Red Cell Distribution Width
SOPs: Standard Operating Procedures
SPSS: Statistical Package for Social Science
WBC: White Blood Cell
